# Efficient Lattice-Based Ring Signature Scheme without Trapdoors for Machine Learning

**DOI:** 10.1155/2022/6547464

**Published:** 2022-09-19

**Authors:** Qing Ye, Yongkang Lang, Zongqu Zhao, Qingqing Chen, Yongli Tang

**Affiliations:** School of Software, Henan Polytechnic University, Jiaozuo 454000, China

## Abstract

Machine learning (ML) and privacy protection are inseparable. On the one hand, ML can be the target of privacy protection; on the other hand, it can also be used as an attack tool for privacy protection. Ring signature (RS) is an effective way for privacy protection in cryptography. In particular, lattice-based RS can still protect the privacy of users even in the presence of quantum computers. However, most current lattice-based RS schemes are based on a strong trapdoor like hash-and-sign, and in such constructions, there is a hidden algebraic structure, that is, added to lattice so that the trapdoor shape is not leaked, which greatly affects the computational efficiency of RS. In this study, utilizing Lyubashevsky collision-resistant hash function over lattice, we construct an RS scheme without trapdoors based on ideal lattice via Fiat‒Shamir with aborts (FSwA) protocol. Regarding security, the proposed scheme satisfies unconditional anonymity against chosen setting attacks (UA-CSA), which is stronger than anonymity against full key exposure (anonymity-FKE), and moreover, our scheme satisfies unforgeability with respect to insider corruption (EU-IC). Regarding computational overhead, compared with other RS schemes that satisfy the same degree of security, our scheme has the highest computational efficiency, the signing and verification time costs of the proposed scheme are obviously better than those of other lattice-based RS schemes without trapdoors, which is more suitable for ML scenarios.

## 1. Introduction

Machine learning (ML) and privacy protection are inextricably linked. On the one hand, ML itself requires privacy protection, i.e., the training datasets and models in ML systems should not be disclosed. On the other hand, ML can also be an attack tool for privacy protection, and how to protect users' sensitive information is a challenging task under the increasingly powerful ML technology.

Ring signature (RS), introduced by Rivest et al. [1] in 2001, is an effective technique for privacy protection in cryptography, which on the one hand enables the recipient of the data to believe that the source of the data is reliable, and on the other hand, obscures the identity of the data owner so that the recipient cannot be sure who is the real owner of the data. In an RS scheme, each user has a public key, and the signer can autonomously collect user's public keys to form a ring without the permission or assistance of other users (where the signer is contained in the set of ring members), and the verifier only knows that the signature was generated under the ring, but is unsure which member's private key is used as the signing key. Due to the anonymity of RS, it is widely used in fields such as e-voting, anonymous membership authentication, and anonymous tip-off.

Since the seminal work of [1], the research on RS has been unprecedentedly active. A general framework for constructing 1-out-of-*n* signature schemes was introduced by Abe et al. [2], which can use different types of keys to construct signatures based on integer decomposition and discrete logarithm problems. After that, many RS schemes along with their associated authentication schemes [3–10] have been proposed. However, the security definitions of these RS schemes are weak, i.e., they have not considered for certain realistic attacks. Bender et al. [11] proposed new definitions of anonymity and the unforgeability of RS to cover these attacks. Bender et al. [11] divided anonymity into three levels according to the degree of security, where the strongest version is anonymity against full key exposure (Anonymity-FKE), i.e., even if adversary is given the private keys of all ring members, the adversary is still unable to guess who is the genuine signer of the given RS. Regarding existential unforgeability, there are also three levels: unforgeability against fixed-ring attacks (EU-FRA), unforgeability against chosen-subring attacks (EU-CSA), and unforgeability with respect to insider corruption (EU-IC). Based on EU-CSA, the strongest EU-IC means the adversary cannot succeed in forging a signature, even if the adversary is allowed to obtain the private keys of ring members via asking a corruption oracle.

Most previous RS schemes [1–7, 9–11] are constructed under the assumptions of classical number theory, which are hardly resistant to quantum attacks [12]. In 1996, Ajtai [13] introduced the algebraic structure of lattice into cryptographic schemes. In the postquantum era, the new cryptosystem based on lattice has become a focus of research due to its merits of high asymptotic efficiency, simple operation, parallelizability, resistance to quantum attacks, and enjoying the average-to-worst reduction. The development of lattice-based provably secure encryption has developed rapidly and has made great progress [14–18], while lattice-based digital signature has experienced a tortuous and bumpy process in the earlier years. First, Goldreich et al. [19] made an attempt at a lattice-based signature, then NTRU signature was proposed by Hoffstein et al. [20], and it was repaired and enhanced in [21, 22]. However, these digital signature schemes [19, 20, 22] on lattice failed to be proved secure under the attacks of [23, 24]. To date, provably secure signature schemes on lattice can be divided into two main branches: schemes with trapdoors and without trapdoors. Digital signature schemes with trapdoors started from the “hash-and-sign” signature scheme constructed by Gentry et al. [25] based on SIS assumption, which is also the first lattice-based signature scheme, that is, provably secure. and Peikert [26] improved the structure of trapdoors, that is, proposed by [27], proposed the concept of *G* trapdoors on lattice, and remarkably improved the computational efficiency of trapdoor generation algorithm on lattice. A digital signature scheme on an ideal lattice was proposed by Ducas and Micciancio [28], and its trapdoor is constructed utilizing the technique of [26]. These trapdoors are deemed to be very suitable for lattice-based signature schemes, but the lattice is added with a hidden algebraic structure, which significantly affects the efficiency of signature schemes and is a payment that has to be considered. Digital signature schemes on lattice without trapdoors are mainly based on Stern's zero-knowledge proofs. Although the Stern-type protocol is powerful, the soundness error of a single execution of the protocol is 2/3. Such protocol needs to be repeated many times so that the soundness error drops to a negligible value, so it is difficult to further improve their efficiency. To the best of our knowledge, only Lyubashevsky signature schemes [29, 30] without trapdoors are constructed not based on the Stern-type protocol, but on the Fiat‒Shamir with aborts (FSwA) protocol, via which a very efficient digital signature scheme on lattice could be constructed.

Similar to a digital signature over lattice, lattice-based (linkable) RS can also be mainly divided into two branches, i.e., with trapdoors and without trapdoors. Lattice-based RS schemes with trapdoors have been extensively studied [31–36]. Notice that these lattice-based RS schemes with trapdoors, although are progressing about storage overhead, cannot always avoid the drawbacks brought by the structure of hash-and-sign, and the computational efficiency cannot be enhanced to a satisfactory level. Lattice-based RS schemes without trapdoors can be classified into membership proofs-based schemes and FSwA-based schemes. Several RS schemes [37–40] based on membership proofs have been proposed. Such RS schemes usually first construct a membership proof which is generally a zero-knowledge proof and then construct an RS scheme based on this proof. The efficiency of these RS schemes directly depends on that of underlying zero-knowledge proofs, which have the advantage that the signature length is usually *O*(log *N*), where *N* is the ring size, whereas the disadvantage is also obvious, i.e., the large and complex zero-knowledge proofs lead to low computational efficiency, and the length of RS could be large when *N* is small. (Linkable) RS schemes [41–44] based on FSwA are rather efficient in terms of computational efficiency, with the drawback that the signature length is common *O*(*N*), yet it is very suitable in small-scale scenarios. For RS on lattice, several schemes [37–40] can satisfy the strongest Anonymity-FKE and EU-IC defined by Bender et al. [11]. In fact, Aguilar Melchor et al. [41] defined stronger anonymity, i.e., unconditional anonymity against chosen setting attacks (UA-CSA), and inspired by Lyubashevsky signature scheme [29], Aguilar Melchor et al. [41] constructed two RS schemes (AM1 and AM2) utilizing lattice-based collision-resistant hash function *h* ∈ *ℋ*(*D*, *D*_*x*_, *m*) [45], both of which can achieve UA-CSA for anonymity; regarding unforgeability, AM1 and AM2 satisfy EU-CSA and EU-IC, respectively. However, we deem that Aguilar Melchor et al. do not make good use of *h* ∈ *ℋ*(*D*, *D*_*x*_, *m*) in transforming Lyubashevsky digital signature [29] into RS, which causes the storage and computational overheads of both schemes of Aguilar Melchor et al. [41] to be large. In this work, via the FSwA protocol, we redesign an RS scheme on lattice without trapdoors using *h* ∈ *ℋ*(*D*, *D*_*x*_, *m*) again, the main contributions are as follows:

1. Different from the RS schemes of Aguilar Melchor et al. [41] on lattice, in our key generation algorithm, the input value of *h* ∈ *ℋ*(*D*, *D*_*x*_, *m*) is taken as a user's private key, the output value of *h* ∈ *ℋ*(*D*, *D*_*x*_, *m*) is taken as a public key, that is, relevant to the private key, which makes the length of a public key is reduced from a polynomial vector of *m* dimensions to a polynomial. Our signing algorithm is designed based on the framework of the RS scheme of Abe et al. [2], which is based on the discrete-log assumption, and the proposed scheme will be more concise and efficient.
2. Under the improved security model of Aguilar Melchor et al. [41], the proposed RS scheme is rigorously proven to be safe. Regarding anonymity, our scheme satisfies the strongest UA-CSA; in terms of unforgeability, our scheme satisfies the strongest EU-IC. And under the random oracle model, the unforgeability of the proposed scheme could be reduced to the approximate shortest vector problem (SVP_*γ*_) over ideal lattice.
3. Finally, with respect to performance and security, the proposed scheme is comprehensively compared with several schemes [37, 38, 40, 41]. The results show that compared with AM1 and AM2 of [41], the storage and computational overhead of our scheme are significantly reduced. In addition, compared with the computational overhead of other schemes, our scheme is remarkably superior, and is more suitable for ML applications.

## 2. Preliminaries

### 2.1. Notations

The symbol [*N*] represents the abbreviation of the set {1,2,…, *N*}. Let *ℝ* be the set of real numbers, *ℝ*^+^ be the set of positive real numbers, and *ℤ* be the set of integers. For a finite set *S*, the symbol *y* ← *S* denotes a random uniform sampling from *S*. The upper-case letter *X* is the random variable denoting a signature, and *X* ← *ℱ* denotes *X* as the output of the signature algorithm *ℱ*. Vectors and matrices are denoted by lower-case (e.g., **x**) and upper-case (e.g., **X**) letters in italic & bold, respectively. In this work, we construct a cryptographic scheme on ring *D* = *ℤ*_*q*_[*x*]/(*x*^*n*^ + 1), where (*x*^*n*^ + 1) is an irreducible polynomial, and all logarithms we use are base 2. *ℤ*_*q*_ is the set of integers modulo *q*, elements in *ℤ*_*q*_ are denoted by integers selected from the interval [−*q* − 1/2, *q* − 1/2], and then the elements in *D* are represented by *n* − 1 degree polynomials whose coefficients are taken from *ℤ*_*q*_. For *a* = ∑_*i*_*f*_*i*_*x*^*i*^ ∈ *D*, the common norms of a are given as follow:

(1) $$\begin{array}{c} l_{1}:{\left\|a\right\|}_{1}=\underset{i}{\sum }\left|f_{i}\right|\text{,}\\ l_{2}:{\left\|a\right\|}_{2}={\left(\underset{i}{\sum }{\left|f_{i}\right|}^{2}\right)}^{1/2}\text{,}\\ l_{\infty }:{\left\|a\right\|}_{\infty }=\max_{i}\left|f_{i}\right|\text{.} \end{array}$$

For a polynomial vector **a**=(*a*_1_, *a*_2_,…, *a*_*m*_), where *a*_1_, *a*_2_,…, *a*_*m*_ ∈ *D*,*m* is a positive integer, and the infinite norm with respect to ***a*** is defined as follows:

(2) $$\begin{array}{c} {\left\|a\right\|}_{\infty }=\max_{i}{\left\|a_{i}\right\|}_{\infty }\text{.} \end{array}$$

Additionally, we will use the following notations:

(3) $$\begin{array}{c} D_{c}=\left\{g\in D:{\left\|g\right\|}_{\infty }\leq 1\right\}\text{,}\\ D_{s}=\left\{g\in D:{\left\|g\right\|}_{\infty }\leq \sqrt{n}\log\;n\right\}\text{,}\\ D_{x}=\left\{g\in D:{\left\|g\right\|}_{\infty }\leq mn^{1.5}\;\log\;n+n\;\log^{2}\;n\right\}\text{,}\\ D_{y}=\left\{g\in D:{\left\|g\right\|}_{\infty }\leq mn^{1.5}\;\log\;n\right\}\text{,}\\ D_{z}=\left\{g\in D:{\left\|g\right\|}_{\infty }\leq mn^{1.5}\;\log\;n-n\;\log^{2}\;n\right\}\text{.} \end{array}$$

The complexity of algorithms is measured using the standard asymptotic notations *ω*, *O*:

(4) $$\begin{array}{c} f\left(n\right)=\omega \left(g\left(n\right)\right)\text{if }\lim_{n⟶\infty }\frac{f\left(n\right)}{g\left(n\right)}=\infty \text{,}\\ f\left(n\right)=O\left(g\left(n\right)\right)\text{if }\lim_{n⟶\infty }\frac{f\left(n\right)}{g\left(n\right)}\neq \infty \text{.} \end{array}$$

Use the symbol $\tilde{O}$ to suppress poly-logarithmic factors, and for example, for any constant *c* and *c*′, *f*(*n*)=*O*(*n*^*c*^ log^*c*′^ *n*), we have $f\left(n\right)=\tilde{O}\left(n^{c}\right)$.

### 2.2. Lattice and Ideal Lattice

Micciancio [46] first proposed the concept of cyclic lattice, which to a certain extent eliminated the drawbacks of big key size and operational inefficiency of cryptographic schemes on Euclidean lattice. Lyubashevsky and Micciancio [45] first proposed the definition of an ideal lattice, which is a lattice with a special algebraic structure and is a generalization of the cyclic lattice. In general, a Euclidean lattice is a subgroup of a group, and an ideal lattice is an ideal of a ring.

**Definition 1 .**

(lattice). Supposing matrix **B** is composed of a set of linearly independent vectors **b**_1_, **b**_2_,…, **b**_*m*_ ∈ *ℤ*^*n*^ , then the integer lattice generated by **B** is defined as:

(5) $$\begin{array}{c} L\left(O\right)=\left\{Bx=\sum x_{i}b_{i}:x\in Z^{m}\right\}\text{,} \end{array}$$

**b**_1_, **b**_2_,…, **b**_*m*_ form a set of bases over *ℒ*(**B**). In short, *ℒ*(**B**) is a discrete additive subgroup in the *n*-dimensional real space *ℝ*^*n*^.

**Definition 2 .**

(*q-*ary lattice). Given a prime q, positive integers m, n, for any matrix **A** ∈ *ℤ*_*q*_^*n*×*m*^, the integer lattice, that is, *m*-dimensional full-rank is described as follows:

(6) $$\begin{array}{c} \Lambda^{⊥}\left(A\right)=\left\{u\in Z^{m}\;s.t.\;Au=0\left(\mathrm{mod}\;q\right)\right\}\text{.} \end{array}$$

**Definition 3 .**

(ideal lattice). Let *f* be a monic irreducible polynomial of degree n, the ideal *I*⊆*ℤ*[*x*]/(*f*), then an integer lattice *ℒ*(**B**)⊆*ℤ*^*n*^ such that *ℒ*(**B**)={*g* mod *f* : *g* ∈ *I*} is called an ideal lattice.

### 2.3. Hardness Assumption

**Definition 4 .**

(R-SIS_*q,m,β*_ problem). Given a randomly chosen vector **a** ∈ *D*^*m*^, find the vector **x** ∈ *D*^*m*^ such that **a**^*T*^ · **x**=0 satisfying 0 < ‖**x**‖_2_ ≤ *β*, where q, m are positive integers, *β* ∈ *ℝ*^+^.

**Definition 5 .**

(SVP_*γ*_). Given a lattice *ℒ*(**B**) and an approximation factor *γ* ≥ 1, find a nonzero vector **v** on lattice such that ‖**v**‖_*∞*_ ≤ *γ*‖**u**‖_*∞*_ holds for any vector **u** ∈ *ℒ*(**B**).

### 2.4. Collision-Resistant Hash Functions and Bounding ‖*ac* mod (*x*^*n*^+1)‖_*∞*_

Lyubashevsky and Micciancio [45] defined a family of collision-resistant hash functions on ideal lattice, which are efficient functions based on the hardness of worst-case lattice problems, and showed that finding collisions for *h* ∈ *ℋ*(*D*, *D*_*x*_, *m*) is at least as hard as solving SVP_*γ*_, where $\gamma =\tilde{O}\left(n^{2}\right)$.

**Definition 6 .**

(family of hash functions). For any integer *m* and set *D*_*x*_, the family of hash functions *ℋ*(*D*, *D*_*x*_, *m*) = {*h*_**a**_ : **a** ∈ *D*^*m*^}, where a hash function *h*_**a**_ : *D*_*x*_^*m*^⟶*D*. On input polynomial vectors **b** = (*b*_1_, *b*_2_,…, *b*_*m*_) ∈ *D*_*x*_^*m*^, then *h*_**a**_(**b**) = **a** · **b** = *a*_1_*b*_1_ + *a*_2_*b*_2_ + ⋯+*a*_*m*_*b*_*m*_. There are two homomorphic properties about *h* ∈ *ℋ*(*D*, *D*_*x*_, *m*) as follows:

(7) $$\begin{array}{c} h\left(x+y\right)=h\left(x\right)+h\left(y\right)\text{,}\\ h\left(xc\right)=h\left(x\right)c\text{,} \end{array}$$

where **x**, **y** ∈ *D*^*m*^, *c* ∈ *D*.

**Definition 7 .**

(collision problem Col(*h*, *D*_*x*_)). Given a function *h* ∈ *ℋ*(*D*, *D*_*x*_, *m*), find two distinct vectors **z**_0_, **z**_1_ ∈ *D*_*x*_ such that *h*(**z**_0_)=*h*(**z**_1_).

The following lemma shows that when *D*_*x*_ is some limited domain (e.g., a set of small norm polynomials), solving the Col(*h*, *D*_*x*_) problem is as hard as solving SVP_*γ*_ in the worst case on the lattice corresponding to the ideal in*D*_*x*_.

**Lemma 1 .**

(see [29]).*Let n be any power of 2, ringD*=*ℤ*_*q*_[*x*]/(*x*^*n*^+1)*, and define the setD*_*x*_={*g* ∈ *D* : ‖*g*‖_*∞*_ ≤ *d*}*, where d is an integer.ℋ*(*D*, *D*_*x*_, *m*)*is a family of hash functions as in *Definition 6* such that m* > log *q*/log 2 *d and q* ≥ 4 *dmn*^1.5^ log *n. For any h* ∈ *ℋ*(*D*, *D*_*x*_, *m*)*, if there exists a polynomial-time algorithm that solves *Col(*h*, *D*_*x*_)* with some non-negligible probability, then there exists a polynomial-time algorithm that can solve *SVP_*γ*_* over ideal lattice, where γ*=16dmn log^2^ *n*.

In addition, we recall the following boundary Lemma to justify the security of the proposed scheme.

**Lemma 2 .**

(Lemma 2.11 in [47] applied to our setting).*Leta* ← *D*_*s*_*, c be the response returned by the random oracleH* (*i.e.,c* ← *D*_*c*_)*, then*

(8) $$\begin{array}{c} \mathrm{Pr}\;\left[{\left\|ac\right\|}_{\infty }\leq n\;\log^{2}\;n\right]\geq 1-4ne^{-\log^{2}\;n/8}=1-n^{-\omega \left(1\right)}\text{.} \end{array}$$

### 2.5. Statistical Distance and Probabilistic Lemma

The difference between two probability distributions can be measured by the statistical distance, and in the security proof of the proposed RS scheme, we complete the proof of anonymity by using it.

**Definition 8 .**

(statistical distance). For random variables *X*_0_ and *X*_1_ that are defined on a countable set *S*, if the set *S* is discrete, then the statistical distance between *X*_0_ and *X*_1_ is described as

(9) $$\begin{array}{c} \Delta \left(X_{0},X_{1}\right)=\frac{1}{2}\underset{x\in S}{\sum }\left|\mathrm{Pr}\;\left[X_{0}=x\right]-\mathrm{Pr}\;\left[X_{1}=x\right]\right|\text{.} \end{array}$$

If the random variables *X*_0_ and *X*_1_ satisfy Δ(*X*_0_, *X*_1_) ≤ *n*^−*ω*(1)^, then *X*_0_ and *X*_1_ are statistically close. Regarding statistical distances, there are the following common properties:

1. Δ(*X*_0_, *X*_1_) ≥ 0;
2. Δ(*X*_0_, *X*_1_)=Δ(*X*_1_, *X*_0_).

In addition, to prove the convergence of our algorithms, the following probabilistic lemma will be used:

**Lemma 3 .**

(Corollary 6.2 in [47]).*For any ***s** ∈ *D*_*s*_^*m*^,

(10) $$\begin{array}{c} {\mathrm{Pr}}_{c\leftarrow D_{c},u\leftarrow D_{y}^{m}}\;\left[sc+u\in D_{z}^{m}\right]=\frac{1}{e}-o\left(1\right)\text{.} \end{array}$$

## 3. Definition of RS and Security Model

### 3.1. Definition of RS

Suppose there exist *N* members in the ring and each member *U*_*i*_(*i* ∈ [*N*]) has a pair of keys, i.e., public key *pk*_*i*_ and private key *sk*_*i*_, a RS scheme can be composed of four polynomial-time algorithms:

1. Setup(1^*λ*^): Taking a security parameter *λ* as input, it outputs the public parameters pp.
2. KeyGen(*pp*): Taking the public parameters pp as input, it generates a pair of keys (*pk*, *sk*), where *pk* and *sk* denote the public and private keys, respectively.
3. Sign(*pp*, *L*_*pk*_, *μ*, *sk*_*π*_): On input public parameters pp, ring *L*_*pk*_, message *μ* and the private key *sk*_*π*_ of the signer *U*_*π*_ (require that its corresponding public key *pk*_*π*_ ∈ *L*_*pk*_), it outputs an RS sig of *U*_*π*_ on the message *μ* under ring *L*_*pk*_.
4. Verify(*pp*, *L*_*pk*_, *μ*, *sig*): On input public parameter pp, ring *L*_*pk*_, message *μ* and RS sig, if sig satisfies the verification conditions, it outputs 1; otherwise, it outputs 0.

### 3.2. Security Model

For the above algorithms, an RS scheme is called to be secure if it satisfies the following definitions: correctness, anonymity, and unforgeability.

**Definition 9 .**

(correctness). If the signer signs honestly, i.e., according to the algorithms in Section 3.1, the Verify algorithm will always output 1 with overwhelming probability, that is, equality (11) holds.

(11) $$\begin{array}{c} \mathrm{Pr}\;\left[\text{Verify}\left(pp,L_{pk},\mu ,sig\right)=1\left|\begin{array}{c} pp\leftarrow \text{Setup}\left(1^{\lambda }\right)\\ \begin{array}{c} \left(pk_{\pi },sk_{\pi }\right)\leftarrow \text{KeyGen}\left(pp\right)\\ pk_{\pi }\in L_{pk} \end{array}\\ \text{sig}\leftarrow \text{Sign}\left(pp,L_{pk},\mu ,sk_{\pi }\right) \end{array}\right.\right]=1-\text{negl}\left(\lambda \right)\text{.} \end{array}$$

Bender et al. [11] proposed the security definitions of RS under different security degrees, among which the highest degree in terms of anonymity is anonymity-FKE. Based on anonymity-FKE, Aguilar Melchor et al. [41] proposed a stronger definition, namely UA-CSA. In this work, the security proof of anonymity is based on the security model of UA-CSA.

Let's define a game between adversary *𝒜* and challenger *S* Under UA-CSA since all secrets are known, the adversary can effectively simulate the signature and corrupt oracles, so these two oracles are no longer provided in the game and the procedure in which *S* generates pp and (*pk*, *sk*) is not required, since they will be generated by the adversary instead. The game is as follows:

- Suppose *l* is the upper bound of ring member size in the system, the adversary submits to a set of public parameters pp, ring *L*_pk_ = {*pk*_1_, *pk*_2_,…, *pk*_*N*_}, two private keys *sk*_*i*_0__, *sk*_*i*_1__, message *μ*, where N, *i*_0_, *i*_1_ ∈ [*l*].
- *S* randomly selects *b* ← {0,1}, invokes Sign(*pp*, *L*_*pk*_, *μ*, *sk*_*i*_*b*__) to generate the signature sig_*i*_*b*__, and returns sig_*i*_*b*__ to *𝒜*.
- Adversary *𝒜* outputs a bit *b*′, and if *b*′ = *b*, then *𝒜* wins the game.

The advantage of winning the game is denoted by,

(12) $$\begin{array}{c} {A\;dv}_{A}^{anon}=\left|\mathrm{Pr}\;\left[b^{\prime }=b\right]-\frac{1}{2}\right|\text{.} \end{array}$$

**Definition 10 .**

(anonymity). An RS scheme satisfies UA-CSA if *A* *dv*_*𝒜*_^anon^ is negligible for any polynomial-time adversary *𝒜*.

Bender et al. [11] consider two cases when defining the strongest unforgeability EU-IC: (1) An adversary can trick some honest user into using the public keys that are adversarially generated to generate a signature. (2) The adversary can adaptively corrupt some ring members and obtain their keys. The strongest EU-IC with respect to RS is depicted by the game between adversary *𝒜* and challenger *𝒜*′ as follows:

(i) Setup phase: Given security parameter *λ*, *𝒜*′ runs the KeyGen algorithm to generate the user's public/private key pairs, and sends public parameters *pp* and the maximum set of user's public keys *S*=(*pk*_*i*_)_*i*∈[*l*]_ to adversary *𝒜*.
(ii) Query phase: *𝒜* is allowed to make adaptive queries to the signature oracle *SO* and the corrupt oracle *CO*.
  - Signing query: *𝒜* submits to *𝒜*′ the set of user's public keys *L*_*pk*_, message *μ* and *π* ∈ [*l*], where *π* is an index such that *pk*_*π*_ ∈ *L*_*pk*_. On receiving (*π*, *μ*, *L*_pk_), *𝒜*′ invokes the Sign algorithm to generate sig and send it to *𝒜*.
  - Corruption query: *𝒜* submits *i*(*i* ∈ [*l*]) to access corrupt oracle *CO*, then *𝒜*′ returns the relevant private key *sk*_*i*_ to *𝒜*.
(iii) Forgery phase: *𝒜* outputs (*μ*^*∗*^, *L*_pk_^*∗*^,sig^*∗*^), and we call *𝒜* gets the triumph if the below conditions are satisfied:
  - Verify(*pp*, *L*_*pk*_^*∗*^, *μ*^*∗*^, sig^*∗*^)=1;
  - *𝒜* never queried for the signature on (*∗*, *μ*^*∗*^, *L*_pk_^*∗*^);
  - *L*_pk_^*∗*^⊆*S*\*C*, where *C* is the set of corrupted users.

The advantage of *𝒜* winning the game is depicted as:

(13) $$\begin{array}{c} {A\;dv}_{A}^{\text{forge}}=\mathrm{Pr}\;\left[Awins\;the\;game\right]\text{.} \end{array}$$

**Definition 11 .**

(unforgeability). A RS scheme is said to satisfy EU-IC if Adv_*A*_^forge^ is negligible for any polynomial-time adversary *𝒜*.

## 4. Lattice-based RS Scheme without Trapdoors

### 4.1. Construction

(1) Setup(1^*λ*^): Given security parameter *λ*, it outputs the public parameter *pp*={*n*, *d*, *q*, *m*, *H*, *h*, *D*_*s*_, *D*_*c*_, *D*_*x*_, *D*_*y*_, *D*_*z*_}, where integer *n* > *λ* and is a power of 2, $d=\sqrt{n}\log\;n$, prime *q* ≥ 4(*mn*^1.5^ log *n*)^2^, integer *m* > log *q*/log 2 *d*, *H* : {0,1}^*∗*^⟶*D*_*c*_ is a cryptographic hash function, *h* is randomly chosen from the family *ℋ*(*D*, *D*_*x*_, *m*), and the other parameters are defined in Section 2.1.
(2) KeyGen(*pp*): Given public parameters pp, it picks **r** ← *D*_*s*_^*m*^, then generates the public key *pk*=*h*(**r**) and sets the private key *sk*=**r**.
(3) Sign(*pp*, *L*_*pk*_, *μ*, *sk*_*π*_): Given public parameters pp, ring *L*_pk_={*pk*_1_, *pk*_2_,…, *pk*_*N*_}, message *μ* ∈ {0,1}^*∗*^ and private key *sk*_*π*_=**r**_*π*_ of member *U*_*π*_, it runs as follows:
  (i) Pick **u** ← *D*_*y*_^*m*^, calculate *c*_(*π* mod *N*)+1_=*H*(*L*_*pk*_, *μ*, *h*(**u**));
  (ii) For *i*=*π*+1,…, *N*, 1,…, *π* − 1:
    - Choose **r**_*z*,*i*_ ← *D*_*z*_^*m*^;
    - Calculate *t*_*i*_=*h*(**r**_*z*,*i*_) − *c*_*i*_*pk*_*i*_, *c*_(*i* mod *N*)+1_=*H*(*L*_pk_, *μ*, *t*_*i*_).
  (iii) Calculate **r**_*z*,*π*_=**u**+*c*_*π*_**r**_*π*_;
  (iv) If **r**_*z*,*π*_ ∉ *D*_*z*_^*m*^, then return to step (i);
  (v) Output the signature sig=(*c*_1_, (**r**_*z*.*i*_)_*i*∈[*N*]_).
(4) Verify(*pp*, *L*_pk,_*μ*, sig): Given public parameters pp, ring *L*_pk_={*pk*_1_, *pk*_2_,…, *pk*_*N*_}, message *μ* ∈ {0,1}^*∗*^ and signature sig=(*c*_1_, (**r**_*z*.*i*_)_*i*∈[*N*]_), it accepts the signature and outputs 1 only if sig satisfies the below conditions, otherwise it rejects the signature and outputs 0.
  - For *i* ∈ [*N*], **r**_*z*,*i*_ ∈ *D*_*z*_^*m*^;
  - For *i* ∈ [*N*], calculate *t*_*i*_=*h*(**r**_*z*,*i*_) − *c*_*i*_*pk*_*i*_, *c*_*i*+1_=*H*(*L*_*pk*_, *μ*, *t*_*i*_), and determine *c*_1_=*H*(*L*_*pk*_, *μ*, *t*_*N*_)=*c*_*N*+1_.

### 4.2. Correctness and Convergence of the Scheme

**Theorem 1 .**

(correctness). *The proposed RS scheme satisfies correctness.*

**Proof**

When sig is a signature generated according to the signature algorithm, then (a) in step (ii) and step (iv) of Sign ensure that (**r**_*z*,*i*_)_*i*∈[*N*]_ are elements in *D*_*z*_^*m*^. In addition, we have the following equations:

(14) $$\begin{array}{c} c_{2}=H\left(L_{\text{pk}},\mu ,t_{1}\right),\text{where }t_{1}=h\left(r_{z,1}\right)-c_{1}pk_{1}\text{,}\\ c_{3}=H\left(L_{\text{pk}},\mu ,t_{2}\right),\text{where }t_{2}=h\left(r_{z,2}\right)-c_{2}pk_{2}\text{,}\\ \vdots \\ c_{\pi }=H\left(L_{\text{pk}},\mu ,t_{\pi -1}\right)\text{,}\\ \text{where }t_{\pi -1}=h\left(r_{z,\pi -1}\right)-c_{\pi -1}pk_{\pi -1}\text{,}\\ \text{since }r_{z,\pi }=u+c_{\pi }r_{\pi }\in D_{z}^{m},\text{we have}\\ c_{\pi +1}=H\left(L_{\text{pk}},\mu ,t_{\pi }\right)=H\left(L_{\text{pk}},\mu ,h\left(r_{z,\pi }\right)-c_{\pi }pk_{\pi }\right)\\ =H\left(L_{\text{pk}},\mu ,h\left(u+c_{\pi }r_{\pi }\right)-c_{\pi }{\text{pk}}_{\pi }\right)\\ =H\left(L_{\text{pk}},\mu ,h\left(u\right)\right),c_{\pi +2}\\ =H\left(L_{\text{pk}},\mu ,t_{\pi +1}\right),\text{where }t_{\pi +1}\\ =h\left(r_{z,\pi +1}\right)-c_{\pi +1}{\text{pk}}_{\pi +1},c_{\pi +3}=H\left(L_{\text{pk}},\mu ,t_{\pi +2}\right)\text{,}\\ \text{where }t_{\pi +2}=h\left(r_{z,\pi +2}\right)-c_{\pi +2}pk_{\pi +2}\text{,}\\ \vdots \\ c_{1}=c_{N+1}=H\left(L_{\text{pk}},\mu ,t_{N}\right),\text{where }t_{N}=h\left(r_{z,N}\right)-c_{N}pk_{N}\text{.} \end{array}$$

To sum up, the polynomial sequence {*c*_1_, *c*_2_,…, *c*_*N*_} in the verification process is equal to that in the signature process, so it must pass the verification of the Verify algorithm, and the proposed scheme is correct.

**Theorem 2 .**

(convergence). *Under the parameter settings of algorithm Setup, the expected runtime of the proposed scheme is*$\tilde{O}\left(n\right)$*, and algorithm Sign is expected to repeat no more than three times.*

**Proof**

The proposed RS scheme is made up of four algorithms: Setup, KeyGen, Sign and Verify The Setup algorithm selects a hash function *h* ∈ *ℋ*(*D*, *D*_*x*_, *m*) (i.e., pick *mn* random numbers from {−(*q* − 1)/2, −(*q* − 1)/2+1,…, (*q* − 1)/2} ), the time for the step is negligible. The step of generating a single user's private/public keys in KeyGen is to randomly select a vector **r** ← *D*_*s*_^*m*^, which simply involves randomly selecting *mn* numbers from the set $\left\{-\sqrt{n}\log\;n,\ldots ,\sqrt{n}\log\;n\right\}\text{,}$ and then calculating *pk*=*h*(**r**), which takes $\tilde{O}\left(n\right)$ time according to Lemma 2.16 from [47]. The Sign algorithm is to randomly selects a vector **u** ← *D*_*y*_^*m*^ and *N* − 1 vectors **r**_*z*,*i*_ ← *D*_*z*_^*m*^, then calculate small polynomial multiplication *c*_*i*_*pk*_*i*_ and hash function *h* ∈ *ℋ*(*D*, *D*_*x*_, *m*)*N* times, and access random oracles *N* times. The time of Sign algorithm running once is $\tilde{O}\left(n\right)$ from [47], but if **r**_*z*,*π*_ ∉ *D*_*z*_^*m*^, then the operations of Sign need to be repeated again.  Lemma 3 states that for any **r** ← *D*_*s*_^*m*^, Pr_**u**←*D*_*y*_^*m*^,*c*←*D*_*s*__ [**u**+*c ***r** ∈ *D*_*z*_^*m*^]=1/*e* − *o*(1). Therefore, we will iterate Sign no more than three times and the runtime of Sign is also $\tilde{O}\left(n\right)$. Finally, the Verify algorithm needs to calculate small polynomial multiplication *c*_*i*_*pk*_*i*_ and function *h* ∈ *ℋ*(*D*, *D*_*x*_, *m*)*N* times, and access random oracles *N* times, thus the running time is also $\tilde{O}\left(n\right)$.

## 5. Security

### 5.1. Anonymity

Before proving the anonymity of the proposed scheme, we first give and prove the following lemma, which shows that for an adversary who has the ability to distinguish two ring signatures based on adversarially-chosen private keys and the corresponding *c*_*π*_ associated with the private keys, the statistical distance between the following two sets of random variables *Y*_0_ and *Y*_1_ is negligible.

Define two sets of random variables *Y*_0_ = (**α**_*i*_ ∈ *D*_*z*_^*m*^;  *i* ∈ [*N*], *β*) and *Y*_1_ = (**α**_*i*_ ∈ *D*_*z*_^*m*^; *i* ∈ [*N*], *β*) that are obtained from the Sign algorithm with input (*pp*, *L*_*pk*_, *μ*, *sk*_*i*_0__) and (*pp*, *L*_*pk*_, *μ*, *sk*_*i*_1__), where the first *N* components of *Y*_*b*_ represent the first *N* outputs (**r**_*z*,*i*_)_*i*∈[*N*]_ of sig_*i*_*b*__, and the (*N*+1)-th component of *Y*_*b*_ represents *c*_*π*_ corresponding to sig_*i*_*b*__, *b* ← {0,1}. In addition, we use *Y*_*b*_^(*i*)^ to represent the *i*-th component of *Y*_*b*_, *i* ∈ [*N* + 1], e.g., *Y*_0_^(*N* + 1)^ represents *c*_*π*_.

**Lemma 4 .**

*If Y*_0_ *and Y*_1_ *are random variables obtained from two legitimate signatures, and these two legitimate signatures are generated by private keys sk*_*i*_0__, *sk*_*i*_1__* which are adversarially-chosen, we have*

(15) $$\begin{array}{c} \Delta \left(Y_{0},Y_{1}\right)=n^{-\omega \left(1\right)}\text{.} \end{array}$$

**Proof**

First define a set *D*_*c*_(*sk*_*i*_0__, *sk*_*i*_1__)={*d* ∈ *D*_*c*_ : ‖*sk*_*i*_0__*d*‖_*∞*_, ‖*sk*_*i*_1__*d*‖_*∞*_ ≤ *n* log^2^ *n*}. According to Lemma 2, it is concluded that almost all elements of *D*_*c*_ are in *D*_*c*_(*sk*_*i*_0__, *sk*_*i*_1__). Even if the private keys *sk*_*i*_0__, *sk*_*i*_1__ ∈ *D*_*s*_, *n* ≥ *λ* are chosen by the adversary, Lemma 2 will also guarantee that |*D*_*c*_(*sk*_*i*_0__, *sk*_*i*_1__)|/|*D*_*c*_|=1 − *n*^−*ω*(1)^, where *n*^−*ω*(1)^ is a negligible function. Then divide the statistical distance Δ(*Y*_0_, *Y*_1_) into two parts, we have formulas (16) and (17).

Next we will discuss Δ(*Y*_0_, *Y*_1_) in two steps, first to prove that formula (16) is negligible (Step 1), and then to prove that formula (17) is equal to zero (Step 2).

(16) $$\begin{array}{c} \Delta \left(Y_{0},Y_{1}\right)=\frac{1}{2}\underset{\alpha_{i}\in D_{s}^{m};i\in \left[N\right],\beta }{\sum }\left|\mathrm{Pr}\;\left[Y_{0}=\left(\alpha_{i};i\in \left[N\right],\beta \right)\right]-\mathrm{Pr}\;\left[Y_{1}=\left(\alpha_{i};i\in \left[N\right],\beta \right)\right]\right|, \end{array}$$

(17) $$\begin{array}{c} +\frac{1}{2}\underset{\alpha_{i}\in D_{s}^{m};i\in \left[N\right],\beta \in D_{c}\left(sk_{i_{0}},sk_{i_{1}}\right)}{\sum }\left|\mathrm{Pr}\;\left[Y_{0}=\left(\alpha_{i};i\in \left[N\right],\beta \right)\right]-\mathrm{Pr}\;\left[Y_{1}=\left(\alpha_{i};i\in \left[N\right],\beta \right)\right]\right|\text{.} \end{array}$$

**Step 1 .**

Considering the case of *β* ∉ *D*_*c*_(*sk*_*i*_0__, *sk*_*i*_1__), generally, since

(18) $$\begin{array}{c} \left(17\right)\leq \frac{1}{2}\underset{\alpha_{i}\in D_{s}^{m};i\in \left[N\right],\beta \notin D_{c}\left(sk_{i_{0}},sk_{i_{1}}\right)}{\sum }\left|\mathrm{Pr}\;\left[Y_{0}=\left(\alpha_{i};i\in \left[N\right],\beta \right)\right]\right|\\ \leq \frac{1}{2}\underset{\beta \notin D_{c}\left(sk_{i_{0}},sk_{i_{1}}\right)}{\sum }\left|\mathrm{Pr}\;\left[Y_{0}^{\left(N+1\right)}=\beta \right]\right|\leq \underset{\beta \notin D_{c}\left(sk_{i_{0}},sk_{i_{1}}\right)}{\sum }\left|\mathrm{Pr}\;\left[Y_{0}^{\left(N+1\right)}=\beta \right]\right|\text{,} \end{array}$$

and since for any *b* ∈ {0,1}, the variable *Y*_*b*_^(*N* + 1)^ is calculated by the hash function *H*(*L*_*pk*_, *μ*, *t*_*π*−1_), where *t*_*π*__−1_ = *h*(**r**_*z*,*π*−1_) − *c*_*π*−1_*pk*_*π*−1_, thus *β* ∈ *D*_*c*_ and the probability that *Y*_*b*_^(*N* + 1)^ is equal to the given value *β* is 1/|*D*_*c*_|. Note that even hash function *h* ∈ *ℋ*(*D*, *D*_*x*_, *m*) is adversarially chosen, it does not affect the probability that *Y*_0_^(*N* + 1)^ is equal to a given *β*. Further, for each *β* ∉ *D*_*c*_(*sk*_*i*_0__, *sk*_*i*_1__), Pr [*Y*_0_^(*N* + 1)^ = *β*] = 1/|*D*_*c*_|, and since almost all elements of *Dc* are in *D*_*c*_(*sk*_*i*_0__, *sk*_*i*_1__) from Lemma 2, it is evident that the probability of *β* ∉ *D*_*c*_(*sk*_*i*_0__, *sk*_*i*_1__) is negligible. And then we get

(19) $$\begin{array}{c} \left(17\right)\leq \underset{\beta \notin D_{c}\left(sk_{i_{0}},sk_{i_{1}}\right)}{\sum }\frac{1}{\left|D_{c}\right|}\\ =\frac{\left|D_{c}\right|-\left|D_{c}\left(sk_{i_{0}},sk_{i_{1}}\right)\right|}{\left|D_{c}\right|}\\ =1-\frac{\left|D_{c}\left(sk_{i_{0}},sk_{i_{1}}\right)\right|}{\left|D_{c}\right|}=n^{-\omega \left(1\right)}\text{.} \end{array}$$

**Step 2 .**

To prove that the value of formula (17) is zero, it is only necessary to prove that each term in formula (17) is zero. Since the last component *Y*_*b*_^(*N*+1)^ of *Y*_*b*_ is derived from a random oracle H, the probability that *Y*_0_^(*N*+1)^ and *Y*_1_^(*N*+1)^ are equal to a given value *β* is the same. Then it is only necessary to prove that the following equation about conditional probabilities holds:

(20) $$\begin{array}{c} \mathrm{Pr}\;\left[\left(Y_{0}^{\left(i\right)};i\in \left[N\right]\right)=\left(\alpha_{i};i\in \left[N\right]\right)\left|Y_{0}^{\left(N+1\right)}=\beta \right.\right]\\ =\mathrm{Pr}\;\left[\left(Y_{1}^{\left(i\right)};i\in \left[N\right]\right)=\left(\alpha_{i};i\in \left[N\right]\right)\left|Y_{1}^{\left(N+1\right)}=\beta \right.\right]\text{.} \end{array}$$

For any *b* ∈ {0,1}, *Y*_*b*_^(*i*)^=**α**_*i*_ ∈ *D*_*z*_^*m*^ if *i* ∈ [*N*]\*i*_*b*_, thus the probability that *Y*_*b*_^(*i*)^ is equal to a given value is 1/|*D*_*z*_^*m*^|; if *i*=*i*_*b*_,

(21) $$\begin{array}{c} \mathrm{Pr}\;\left[Y_{b}^{\left(i_{b}\right)}=\alpha_{i_{b}}\right]=\mathrm{Pr}\;\left[u_{b}+sk_{i_{b}}\beta =\alpha_{i_{b}}\right]\\ =\mathrm{Pr}\;\left[u_{b}=\alpha_{i_{b}}-sk_{i_{b}}\beta \right]\text{.} \end{array}$$

Since*β* ∈ *D*_*c*_(*sk*_*i*_0__, *sk*_*i*_1__), **α**_*i*_*b*__ ∈ *D*_*z*_^*m*^, we have ‖*sk*_*i*_0__*β*‖_*∞*_, ‖*sk*_*i*_1__*β*‖_*∞*_ ≤ *n* log^2^ *n*, ‖**α**_*i*_*b*__‖_*∞*_ ≤ *mn*^1.5^ log *n* − *n* log^2^ *n*, then ‖**α**_*i*_0__ − *sk*_*i*_0__*β*‖_*∞*_ = ‖**α**_*i*_1__ − *sk*_*i*_1__*β*‖_*∞*_ ≤ *mn*^1.5^ log *n*. Thus, the values of both **α**_*i*_0__ − *sk*_*i*_0__*β* and **α**_*i*_1__ − *sk*_*i*_1__*β* belong to the set *D*_*y*_^*m*^. And since **u**_0_, **u**_1_ ← *D*_*y*_^*m*^, Pr [**u**_0_ = **α**_*i*_0__ − *sk*_*i*_0__*β*] = Pr [**u**_1_ = **α**_*i*_1__ − sk_*π*_1__*β*] = 1/|*D*_*y*_^*m*^|.

In summary, for any *b* ∈ {0,1},

(22) $$\begin{array}{c} \mathrm{Pr}\;\left[Y_{b}^{\left(i\right)}=\alpha_{i}\left|Y_{b}^{\left(N+1\right)}=\beta \right.\right]=\left\{\begin{array}{cc} \frac{1}{\left|D_{z}^{m}\right|}\text{,} & i\in \left[N\right]\backslash i_{b}\text{,}\\ \frac{1}{\left|D_{y}^{m}\right|}\text{,} & i=i_{b}\text{.} \end{array}\right. \end{array}$$

Therefore, it can be proved that equation (20) holds, i.e., the proof of the lemma is completed.

Suppose that in the game of anonymity in Section 3.2, pp are adversarially chosen according to the Setup algorithm, *pk*_*i*_0__ and *pk*_*i*_1__ are adversarially generated according to the KeyGen algorithm, message *μ* and ring *L*_pk_ are also chosen by adversary. The challenger chooses *b* ← {0,1} and invokes Sign(*pp*, *L*_*pk*_, *μ*, *sk*_*b*_) to generate a signature, and give it to the adversary. Define the random variable *X*_*b*,pp, sk_*i*_*b*__,*μ*,L_pk__ to represent the signature generated by the challenger, and the following theorem shows that the statistical distance between *X*_0,pp, sk_*i*_0__,*μ*,L_pk__ and *X*_1,pp, sk_*i*_1__,*μ*,L_pk__ is negligible.

**Theorem 3 .**

(anonymity). *The proposed scheme satisfies UA-CSA, i.e., for the adversary in*Definition 10,*X*_*b*,pp, sk_*i*_*b*__,*μ*,*L*_pk__ ← Sign(*pp*, *L*_*pk*_, *μ*, *sk*_*i*_*b*__), *b* ∈ {0,1}*, we have*

(23) $$\begin{array}{c} \Delta \left(X_{0,pp,sk_{i_{0}},\mu ,L_{pk}},X_{1,pp,sk_{i_{1}},\mu ,L_{pk}}\right)=n^{-\omega \left(1\right)}\text{.} \end{array}$$

**Proof**

Regarding *X*_*b*,pp, sk_*i*_*b*__,*μ*,*L*_pk__, it is known from the signing process, **r**_*z*,*i*_ ← *D*_*z*_^*m*^ if *i* ≠ *i*_*b*_; **r**_*z*,*i*_=**u**+*c*_*i*_**r**_*i*_ if *i*=*i*_*b*_. By Lemma 3 and Theorem 2, we know that **r**_*z*,*i*_ is indistinguishable from a randomly selected vector in *D*_*z*_^*m*^. And since the first component *c*_1_ in *X*_*b*,pp, sk_*i*_*b*__,*μ*,*L*_pk__ is from a random oracle, the statistical distance Δ(*X*_0,*pp*,*sk*_*i*_0__,*μ*,*L*_*pk*__, *X*_1,*pp*,*sk*_*i*_1__,*μ*,*L*_*pk*__)=*n*^−*ω*(1)^ for an ordinary adversary *𝒜*. However, for an UA-CSA adversary, obviously, such an analysis is not rigorous enough. The following will focus on the verification process, whether the adversary can distinguish the two signatures based on adversarially-chosen private keys and *c*_*π*_ associated with the private keys.

Since the first component *c*_1_ of *X*_*b*,pp, sk_*i*_*b*__,*μ*,*L*_pk__ is obtained by accessing a random oracle, and it is not directly related to the associated private key, thus the discussion with respect to *c*_1_ is not necessary. We only need to prove that for an UA-CSA adversary, the statistical distance between the defined *Y*_0_, *Y*_1_ is negligible. By Lemma 4 it is known that Δ(*Y*_0_, *Y*_1_)=*n*^−*ω*(1)^, thus for an UA-CSA adversary, the probability of winning the anonymity game is also negligible. That completes the proof.

### 5.2. Unforgeability

Before proving unforgeability, the following lemma is first given and proved.

**Lemma 5 .**

*Let* **r**, **r**′ ← *D*_*s*_* , ***v**, **v**′ ← *D*_*z*_* , c*, *c*′ ← *D*_*c*_* and c* ≠ *c*′* , if ***v** − *c ***r**=**v**′ − *c*′**r*** , ***r** ≠ **r**′* , then the following inequality holds*:

(24) $$\begin{array}{c} v-cr^{\prime }\neq v^{\prime }-c^{\prime }r^{\prime }\text{.} \end{array}$$

**Proof**

Assume that both **v** − *c ***r** = **v**′ − *c*′**r** and **v** − *c ***r**′ = **v**′ − *c*′**r**′ hold. By subtracting the two equations, we have (**r**′ − **r**)(*c*′ − *c*) = 0. Since the equation holds in ring *ℤ*_*q*_[*x*]/(*x*^*n*^ + 1), it does not directly deduce that **r**′ = **r** or *c*′ = *c* holds. Due to ${\left\|r^{\prime }\right\|}_{\infty },{\left\|r\right\|}_{\infty }\leq \sqrt{n}\log\;n$, ‖*c*′‖_*∞*_, ‖*c*‖_*∞*_ ≤ 1, then the absolute values of the coefficients of **r**′ − **r** and *c*′ − *c* are no more than $2\sqrt{n}\log\;n$ and 2, respectively. When **r**′ − **r** is multiplied by *c*′ − *c* in ring *ℤ*[*x*]/(*x*^*n*^ + 1), the absolute value of the coefficients of (**r**′ − **r**)(*c*′ − *c*) is no more than 4*n*^1.5^ log *n*. Since *q* ≫ 4*n*^1.5^ log *n*, if (**r**′ − **r**)(*c*′ − *c*) = 0 holds in ring *ℤ*_*q*_[*x*]/(*x*^*n*^ + 1), then it must also hold in ring *ℤ*[*x*]/(*x*^*n*^ + 1). And since *c* ≠ *c*′, then it must have **r**′ = **r**, which is contradictory to the assumption, thus the lemma is proved.

**Theorem 4 .**

(unforgeability). *Under the random oracle model, if there exists a polynomial-time adversary 𝒜 who can validly forge a RS signature about the proposed scheme with probability ε, then for a random-chosen h* ∈ *ℋ*(*D*, *D*_*x*_, *m*)*, there is a polynomial-time algorithm 𝒜*′* that can obtain a solution to *Col(*h*, *D*_*x*_)* with probability at least ε*^2^/2(*ψ*+3*Nζ*)*, where N, ζ and ψ are the number of ring members, the maximum times that 𝒜 accesses SO and directly accesses HO, respectively.*

**Proof**

Suppose there exists an EU-IC adversary *𝒜* who can validly forge a signature against the proposed scheme with non-negligible probability *ε*, then there exists a challenger *𝒜*′ who can solve Col(*h*, *D*_*x*_) with non-negligible probability *ε*′.

Suppose the number of maximum ring members in the system is *l*=*l*(*λ*), and given a hash function family *ℋ*(*D*, *D*_*x*_, *m*), *𝒜*′ obtains an instance (*h*) from the Col(*h*, *D*_*x*_) oracle as an input. *𝒜*′ maintains two lists *L*_1_ and *L*_2_, which are initialized to be null. For *i* ∈ [*l*], *𝒜*′ honestly runs the Keygen algorithm to generate the key pair (*pk*_*i*_, *sk*_*i*_), and stores the tuple (*i*, *pk*_*i*_, *sk*_*i*_) in list *L*_1_. *𝒜*′ gives the public key set *S*={*pk*_1_, *pk*_2_,…, *pk*_*l*_} to *𝒜*, and then *𝒜*′ simulates oracles and responds to the queries from *𝒜* in the following manner:

Hash query *HO*: *𝒜* submits a set of ring members *L*_pk_=(*pk*_1_, *pk*_2_,…, *pk*_*N*_) ⊂ *S*, message *μ* ∈ {0,1}^*∗*^ and *t*_*i*_ ∈ *D*, *𝒜*′ inquires the list *L*_2_, and if the tuple (*L*_*pk*_, *μ*, *t*_*i*_, *c*_*i*+1_) exists, *𝒜*′ returns *c*_*i*+1_ to *𝒜*. Otherwise, *𝒜*′ randomly selects *c*_*i*+1_ ∈ *D*_*c*_ and returns it to *𝒜*, and adds (*L*_*pk*_, *μ*, *t*_*i*_, *c*_*i*+1_) to the list *L*_2_.

Signing query *SO*: *𝒜* submits an index *π*, a message *μ* ∈ {0,1}^*∗*^ and a set of ring members *L*_pk_=(*pk*_1_, *pk*_2_,…, *pk*_*N*_) (which may contain some public keys generated by *𝒜* in an arbitrary way). Note that since *𝒜*′ knows the private keys of all members in set *S*, and in general, *𝒜* will not query signatures about a user outside *S*, which is meaningless. *𝒜*′ responds to this query by honestly running the Sign algorithm.

Corruption query *CO*: *𝒜* can make corruption query about any user *U*_*i*_ (*i* ∈ [*l*]). If *𝒜* makes a query on (*i*, *pk*_*i*_), *𝒜*′ first obtains the tuple (*i*, *pk*_*i*_, *sk*_*i*_) by looking for the list *L*_1_, and then returns sk_*i*_ to *𝒜*.

Forgery phase: Suppose that after finishing the above queries, *𝒜* outputs a valid forgery (*μ*^*∗*^, *L*_*pk*_^*∗*^, sig^*∗*^) with non-negligible probability, *ε* and *𝒜* did not ask for any signature on (*∗*, *μ*^*∗*^, *L*_*pk*_^*∗*^), where *L*_pk_^*∗*^⊆*S*\*C* and *C* is the set of corrupted users.

*Analysis*. Define *p* as the maximum times *HO* is queried during *𝒜*'s attack. By Theorem 2, we know that it takes at most 3*NHO* queries to produce a RS, and since *𝒜* can make *SO* queries at most *ζ* times, the value of *p* is at most *ψ*+3*Nζ*. Suppose that sig^*∗*^=(*c*_1_^*∗*^, (**r**_*z*,*i*_^*∗*^)_*i*∈[*N*]_) can pass the verification of Verify, then in general, it can be assumed that for some *j*, we have *H*(*L*_*pk*_^*∗*^, *μ*^*∗*^, *h*(**r**_*z*,*j*_^*∗*^) − *c*_*j*_^*∗*^*pk*_*j*_^*∗*^)=*c*_*j*+1_^*∗*^.

If *𝒜* did not query *HO* on (*L*_*pk*_^*∗*^, *μ*^*∗*^, *h*(**r**_*z*,*j*_^*∗*^) − *c*_*j*_^*∗*^*pk*_*j*_^*∗*^), then the probability of producing *c*_*j*+1_^*∗*^ such that *H*(*L*_*pk*_^*∗*^, *μ*^*∗*^, *h*(**r**_*z*,*j*_^*∗*^) − *c*_*j*_^*∗*^*pk*_*j*_^*∗*^)=*c*_*j*+1_^*∗*^ is only 1/|*D*_*c*_|. Hence, the probability that *c*_*j*+1_^*∗*^ ∈ {*s*_1_, *s*_2_,…, *s*_*p*_} is 1 − 1/|*D*_*c*_|, where *s*_1_, *s*_2_,…, *s*_*p*_ ← *D*_*c*_ is the sequence of return values from *HO*. Further, the probability that *𝒜* succeeds in a forgery and *c*_*j*+1_^*∗*^ ∈ {*s*_1_, *s*_2_,…, *s*_*p*_} is at least *ε* − 1/|*D*_*c*_|. Suppose *c*_*j*+1_^*∗*^=*s*_*j*_, then there are two cases: (1) *s*_*j*_ is a response to *SO* query (Case 1); (2) *s*_*j*_ is a response to *HO* query (Case 2). We first analyze Case 1.

**Case 1 .**

Suppose *c*_*j*+1_^*∗*^ appears during signing query, and suppose that the corresponding response *𝒜* obtains is the signature sig=(*c*_1_, (**r**_*z*,*i*_)_*i*∈[*N*]_) (i.e., *c*_*j*+1_^*∗*^=*H*(*L*_*pk*_, *μ*, *h*(**r**_*z*,*j*_) − *c*_*j*_*pk*_*j*_)). Then compare the signature forged by *𝒜* with the legitimate signature sig=(*c*_1_, (**r**_*z*,*i*_)_*i*∈[*N*]_), we have

(25) $$\begin{array}{c} c_{j+1}^{*}=H\left(L_{\text{pk}}^{*},\mu^{*},h\left(r_{z,j}^{*}\right)-c_{j}^{*}{\text{pk}}_{j}^{*}\right)=H\left(L_{pk},\mu ,h\left(r_{z,j}\right)-c_{j}pk_{j}\right)\text{.} \end{array}$$

Then either *L*_pk_^*∗*^ ≠ *L*_pk_ or *μ*^*∗*^ ≠ *μ* (or **r**_*z*,*j*_^*∗*^ ≠ **r**_*z*,*j*_), since if all three equations hold, this means the adversary simply outputs a signature that has already been asked. If at least one of *L*_pk_^*∗*^ ≠ *L*_pk_ and *μ*^*∗*^ ≠ *μ* holds, it implies that a collision has occurred in *H*. Hence there is *L*_pk_^*∗*^=*L*_pk_, *μ*^*∗*^=*μ* and **r**_*z*,*j*_^*∗*^ ≠ **r**_*z*,*j*_ with overwhelming probability, that is, *H*(*L*_pk_^*∗*^, *μ*^*∗*^, *h*(**r**_*z*,*j*_^*∗*^) − *c*_*j*_^*∗*^pk_*j*_^*∗*^)=*c*_*j*+1_^*∗*^=*H*(*L*_pk_^*∗*^, *μ*^*∗*^, *h*(**r**_*z*,*j*_) − *c*_*j*_^*∗*^pk_*j*_^*∗*^). Therefore either *h*(**r**_*z*,*j*_^*∗*^) − *c*_*j*_^*∗*^pk_*j*_^*∗*^ ≠ *h*(**r**_*z*,*j*_) − *c*_*j*_^*∗*^pk_*j*_^*∗*^, which implies there is a collision in *H*, or *h*(**r**_*z*,*j*_^*∗*^) − *c*_*j*_^*∗*^*pk*_*j*_^*∗*^=*h*(**r**_*z*,*j*_) − *c*_*j*_^*∗*^*pk*_*j*_^*∗*^, which implies there is *h*(**r**_*z*,*j*_^*∗*^)=*h*(**r**_*z*,*j*_). Then we find a collision for *h*, and since **r**_*z*,*j*_, **r**_*z*,*j*_^*∗*^ ∈ *D*_*z*_^*m*^ ⊂ *D*_*x*_^*m*^, we find a solution to Col(*h*, *D*_*x*_). Note that after *HO* is queried *p* times, the probability that a collision happens in *H* is no more than *p*/|*D*_*c*_|, therefore, the probability of *𝒜* outputting a forgery as a solution to Col(*h*, *D*_*x*_) is at least,

(26) $$\begin{array}{c} \frac{\varepsilon -p}{\left|D_{c}\right|}\text{.} \end{array}$$

**Case 2 .**

The next is to consider the case that *s*_*j*_ is obtained by *𝒜* calling *HO*. In this scenario, suppose that *𝒜*′ runs *𝒜* with some randomness and *𝒜*′ obtains a valid forgery sig^*∗*^ = (*c*_1_^*∗*^, (**r**_*z*,*i*_^*∗*^)_*i*∈[*N*]_) of *μ*^*∗*^. Suppose that *s*_1_,…, *s*_*p*_ ← *D*_*c*_ is the sequence of response values from *HO* during this forgery. Then generate fresh random elements *s*_*j*_′,…, *s*_*p*_′ ← *D*_*c*_, and *𝒜*′ runs *𝒜* with the same randomness and responds to *𝒜*'s*HO* query with *s*_1_,…, *s*_*j*−1_, *s*_*j*_′,…, *s*_*p*_′, by forking lemma from [48], *𝒜*′ will obtain another forged signature sig′ = (*c*_1_′, (**r**_*z*,*i*_)_*i*∈[*N*]_) on message *μ*^*∗*^ and *s*_*j*_ ≠ *s*_*j*_′ with probability at least(*ε* − 1/|*D*_*c*_|)((*ε* − 1/|*D*_*c*_|)/(*ψ* + 3*Nζ*) − 1/|*D*_*c*_|). Since the input of *HO* query is the same in both forgeries, we have *h*(**r**_*z*,*j*_^*∗*^) − *c*_*j*_^*∗*^pk_*j*_^*∗*^ = *h*(**r**_*z*,*j*_′ − *c*_*j*_′pk_*j*_^*∗*^). By the homomorphic properties of *h* in Definition 6, it follows that *h*(**r**_*z*,*j*_^*∗*^ − *c*_*j*_^*∗*^**r**_*j*_^*∗*^) = *h*(**r**_*z*,*j*_′ − *c*_*j*_′**r**_*j*_^*∗*^). If **r**_*z*,*j*_^*∗*^ − *c*_*j*_^*∗*^**r**_*j*_^*∗*^ ≠ **r**_*z*,*j*_′ − *c*_*j*_′**r**_*j*_^*∗*^ and **r**_*z*,*j*_^*∗*^ − *c*_*j*_^*∗*^**r**_*j*_^*∗*^, **r**_*z*,*j*_′ − *c*_*j*_′**r**_*j*_^*∗*^ ∈ *D*_*x*_^*m*^, then *𝒜*′ finds a collision for *h*.

Next, we analyze the probability of **r**_*z*,*j*_^*∗*^ − *c*_*j*_^*∗*^**r**_*j*_^*∗*^ ≠ **r**_*z*,*j*_′ − *c*_*j*_′**r**_*j*_^*∗*^. By Lemma 5.2 in [47], we know that there is a distinct private key **r**_*j*_′ such that pk_*j*_=*h*(**r**_*j*_′)=*h*(**r**_*j*_^*∗*^) with overwhelming probability. And by Lemma 5, if the private key **r**_*j*_^*∗*^ satisfies **r**_*z*,*j*_^*∗*^ − *c*_*j*_^*∗*^**r**_*j*_^*∗*^=**r**_*z*,*j*_′ − *c*_*j*_′**r**_*j*_^*∗*^, then for another private key **r**_*j*_′, we have **r**_*z*,*j*_^*∗*^ − *c*_*j*_^*∗*^**r**_*j*_′ ≠ **r**_*z*,*j*_′ − *c*_*j*_′**r**_*j*_′. By Theorem 6.5 from [47], it is statistically impossible to distinguish which one of **r**_*j*_′ and **r**_*j*_^*∗*^ is used by the signer, and thus the probability of **r**_*z*,*j*_^*∗*^ − *c*_*j*_^*∗*^**r**_*j*_^*∗*^ ≠ **r**_*z*,*j*_′ − *c*_*j*_′**r**_*j*_^*∗*^ is at least 1/2 − *n*^−*ω*(1)^. And for any response from *HO*, *s*_*i*_ ∈ *D*_*c*_, then by Lemma 2, we have ‖**r**_*z*,*j*_^*∗*^ − *c*_*j*_^*∗*^**r**_*j*_^*∗*^‖_*∞*_, ‖**r**_*z*,*j*_′ − *c*_*j*_′**r**_*j*_^*∗*^‖_*∞*_ ≤ *mn*^1.5^ log *n*, i.e., **r**_*z*,*j*_^*∗*^ − *c*_*j*_^*∗*^**r**_*j*_^*∗*^, **r**_*z*,*j*_′ − *c*_*j*_′**r**_*j*_^*∗*^ ∈ *D*_*y*_^*m*^ ⊂ *D*_*x*_^*m*^, which implies that *𝒜*′ finds a solution to Col(*h*, *D*_*x*_).

From the above analysis, we know that the probability of *𝒜*′ breaking Col(*h*, *D*_*x*_) via *𝒜* calling *SO* is at least *ε* − *p*/|*D*_*c*_|, while the probability of *𝒜*′ breaking Col(*h*, *D*_*x*_) via *𝒜* calling *HO* is at least,

(27) $$\begin{array}{c} \left(\frac{1}{2}-n^{-\omega \left(1\right)}\right)\left(\varepsilon -\frac{1}{\left|D_{c}\right|}\right)\left(\frac{\varepsilon -1/\left|D_{c}\right|}{\psi +3N\zeta }-\frac{1}{\left|D_{c}\right|}\right)\text{.} \end{array}$$

By comparing formulas (26) and (27), we have the inequality (26) > (27). Thus, the probability of *𝒜*′ running *𝒜* to solve Col(*h*, *D*_*x*_) successfully is,

(28) $$\begin{array}{c} \varepsilon^{\prime }\geq \left(\frac{1}{2}-n^{-\omega \left(1\right)}\right)\left(\varepsilon -\frac{1}{\left|D_{c}\right|}\right)\left(\frac{\varepsilon -1/\left|D_{c}\right|}{\psi +3N\zeta }-\frac{1}{\left|D_{c}\right|}\right)\text{.} \end{array}$$

## 6. Discussion

From Theorem 4, it is known that if an adversary succeeds in forging an RS against the proposed scheme, then the challenger can find a collision for a randomly chosen hash function *h*_**a**_(*∗*) in *ℋ*(*D*, *D*_*x*_, *m*). From [29], solving Col(*h*, *D*_*x*_) is equivalent to finding a vector **u** ∈ Λ_*q*_^⊥^(**Α**) on lattice Λ_*q*_^⊥^(**Α**) = {**u** ∈ *ℤ*^*mn*^ s.t. **Au** = 0(mod *q*)} satisfying ‖**u**‖_*∞*_ ≤ 2(*mn*^1.5^ log *n* + *n* log^2^ *n*) where **Α** = [*Rot*(*a*_1_)‖*Rot*(*a*_2_)‖…‖*Rot*(*a*_*m*_)] ∈ *ℤ*_*q*_^*n*×*nm*^, *a*_*i*_ is the *i*-th component of ***a***, *i* ∈ [*m*].

It was shown in [49] that in a reasonable amount of time, the algorithm for finding short vectors on a random lattice will generate a vector no less than 1.01^*n*^ times the shortest vector over the lattice. Furthermore, based on the experiments of [49], Micciancio and Regev [50] conduct experiments on lattices very similar to Λ_*q*_^⊥^(**Α**), and proves that the length of the shortest vector, which can be found on Λ_*q*_^⊥^(**Α**) by using the well-known lattice reduction algorithm, is ${\left\|z\right\|}_{2}\geq \min\;\left\{q,2^{2\sqrt{n\log q\log1.01}}\right\}$ and the corresponding ‖**z**‖_*∞*_ at least,

(29) $$\begin{array}{c} \min\;\left\{q,2^{2\sqrt{n\;\log\;q\;\log\;1.01}}\cdot {\left(\frac{n\;\log\;q}{\log\;1.01}\right)}^{-1/4}\right\}\text{.} \end{array}$$

Therefore, in order to make Col(*h*, *D*_*x*_) be intractable, we should set the parameters in such a manner that requires 2(*mn*^1.5^ log *n*+*n* log^2^ *n*) to be smaller than formula (29). For example, *n*=512, *q* ≈ 2^41^, then calculate 2(*mn*^1.5^ log *n*+*n* log^2^ *n*) ≈ 2^20.1^, while the infinite norm of the shortest vector ‖**z**‖_*∞*_ that can be found by formula (29) is around 2^29.6^.  Table 1 gives several sets of parameter settings and security levels for our scheme.

## 7. Efficiency

In this section, in respect of efficiency and security, we compare our scheme with five lattice-based RS schemes without trapdoors: the RS schemes of [37, 38, 40] and the two RS schemes AM1 and AM2 of [41]. The comparison results of storage overhead and computational overhead are listed in Table 2 and 3, respectively. *λ* is a security parameter, *n* > *λ* is a power of 2, *N* is the number of ring members, and *q* is a large prime number. In estimating the computational efficiency of each scheme, we mainly focus on the relatively time-consuming operations, such as polynomial inversion and polynomial-polynomial multiplication, while ignoring the less time-consuming operations such as polynomial-polynomial addition and cryptographic hash operation *H*. The notation *T*_poly−inv_ denotes the computational overhead of running polynomial-polynomial multiplication in *D* once, *T*_poly−mul_ denotes the computational overhead of running polynomial inversion once, normally *T*_poly−inv_ > *T*_poly−mul_. *T*_*v*_ denotes the time spent in performing *λ* times sanity checks of parameters in the signing phase of AM2. *T*_*c*_ denotes the computational overhead of selecting a compliant signing key in the signing phase of AM2.

From Table 2, the signature size of [37, 38, 40] is *O*(log *N*), while the signature size of AM1, AM2 and ours is *O*(*N*). Without loss of generality, $n\geq \sqrt{n}\log\;n$, thus compared with AM1, AM2, our scheme has smaller public/private key size and signature size.

These schemes [37, 38, 40] all use complex and bloated zero-knowledge proofs, and it is very difficult for us to calculate their computational overheads. Among these schemes [37, 38, 40], only the relatively concise scheme of [38] gives its computational overheads analysis. Therefore, we only compare the computational cost of [38, 41] and our scheme. We adopt the calculation method of efficiency in [38], and reduce the operations of KeyGen, Sign and Verify to polynomial-polynomial multiplication operations. To make the comparison results more intuitive, the parameters of [38, 41] and ours are set under the same security level *λ*=128. Here, *n* in [38], AM, and ours are set to 256, 128, and 512, respectively; *m* in [38], AM, and ours are set to 15, 21, and 5, respectively. In terms of KeyGen, Sign and Verify, our scheme obviously has the smallest computational cost as shown in Tables 3 and 4.

**Figure 1:**
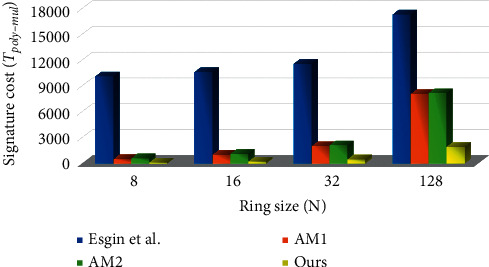
Comparison of computational overhead in the signing phase.

**Figure 2:**
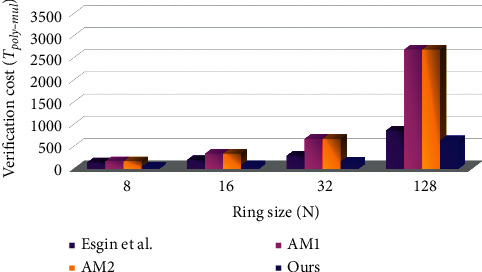
Comparison of computational overhead in the verification phase.

To make the comparison results more intuitive, Figures 1 and 2 show the comparison between [38, 41] and ours in terms of computational overhead under different ring sizes.

## 8. Conclusions

Although lattice-based RS schemes are resistant to attacks by quantum computers, there still exists a big gap between them and the schemes that are based on traditional number-theoretic assumptions with respect to computational efficiency. To further boost the computational efficiency of RS on lattice, this work constructs a lattice-based RS scheme without trapdoors under the random oracle model. Based on a collision-resistant hash function on ideal lattice, our scheme is designed via the FSwA protocol. Our scheme avoids the use of trapdoors or sampling techniques that have high computational overhead and does not involve complex zero-knowledge proofs which are usually used in RS schemes without trapdoors. The proposed scheme is more concise and efficient. Meanwhile, in terms of anonymity and unforgeability, our scheme is proven to satisfy the strongest UA-CSA and EU-IC, respectively. Next, we plan to investigate NTRU lattice-based RS scheme without trapdoors, which will be the first combination of NTRU lattice and RS without trapdoors.

## Figures and Tables

**Table 1:** Parameter setting for our scheme.

Parameter	*n*	*q*(≈)	*m*	The vector length needed to break RS	The shortest vector length that can be found	Security level (bits)
Recommended choice	128	2^79^	_16_	2^18.4^	2^19.2^	80
	256	2^43^	8	2^19.1^	2^20.3^	80
	512	2^41^	_5_	2^20.1^	2^29.6^	128
	1024	2^73^	_8_	2^22.4^	2^59.9^	192

**Table 2:** Comparison of storage overhead and security.

Scheme	Public key size	Private key size	Signature size	UA-CSA	EU-IC
[37]	*O*(*n* log *q*)	*O*(*n* log *n*)	*O*(log (*Nn*)*n* log^2^ *n*)	Yes	Yes
[38]	*O*(*n* log *q*)	*O*(*n*)	*O*(log^5^ *Nn* log^2^ *n*)	Yes	Yes
[40]	*O*(*n* log *q*)	*O*(*n*)	*O*(log *Nn* log (*nq*))	Yes	Yes
AM1 of [41]	*O*(*n* log *n* log *q*)	*O*(*n* log *n*)	*O*(*Nn* log *n* log (*n*^1.5^ log^2^ *n*))	Yes	No
AM2 of [41]	*O*(*λn* log *n* log *q*)	*O*(*λn* log *n*)	*O*(*Nn* log *n* log (*n*^1.5^ log^2^ *n*))	Yes	Yes
Ours	*O*(*n* log *q*)	$O\left(n\;\log\;\left(\sqrt{n}\log\;n\right)\right)$	*O*(*Nn* log (*n*^1.5^ log *n*))	Yes	Yes

**Table 3:** Comparison of computational overhead at security level *λ*=128.

Scheme	Key generation cost	Signature cost	Verification cost
[38]	438*T*_poly−mul_	60*N*+9660*T*_poly−mul_	6*N*+96*T*_poly−mul_
AM1 of [41]	21*T*_poly−mul_+*T*_poly−inv_	63*NT*_poly−mul_	21*NT*_poly−mul_
AM2 of [41]	*λ*(21*T*_poly−mul_+*T*_poly−inv_)/2	63*NT*_poly−mul_+*T*_*v*_+*T*_*c*_	21*NT*_poly−mul_
Ours	5*T*_*poly*−*mul*_	15*NT*_poly−mul_	5*NT*_poly−mul_

**Table 4:** Comparison of approximate computational costs under ring size *N*=2^7^.

Scheme	Key generation cost	Signature cost	Verification cost
[38]	438*T*_poly−mul_	17340*T*_poly−mul_	864*T*_poly−mul_
AM1 of [41]	21*T*_poly−mul_+*T*_poly−inv_	8064*T*_poly−mul_	2688*T*_poly−mul_
AM2 of [41]	*λ*(21*T*_poly−mul_+*T*_poly−inv_)/2	8064*T*_poly−mul_+*T*_*v*_+*T*_*c*_	2688*T*_poly−mul_
Ours	5*T*_poly−mul_	1920*T*_poly−mul_	640*T*_poly−mul_

## Data Availability

The figures and tables used to support the findings of this study are included in the article.
